# Using Twitter Data for Cohort Studies of Drug Safety in Pregnancy: Proof-of-concept With β-Blockers

**DOI:** 10.2196/36771

**Published:** 2022-06-30

**Authors:** Ari Z Klein, Karen O'Connor, Lisa D Levine, Graciela Gonzalez-Hernandez

**Affiliations:** 1 Department of Biostatistics, Epidemiology, and Informatics Perelman School of Medicine University of Pennsylvania Philadelphia, PA United States; 2 Department of Obstetrics and Gynecology Perelman School of Medicine University of Pennsylvania Philadelphia, PA United States; 3 Department of Computational Biomedicine Cedars-Sinai Medical Center Los Angeles, CA United States

**Keywords:** natural language processing, social media, data mining, pregnancy, pharmacoepidemiology

## Abstract

**Background:**

Despite the fact that medication is taken during more than 90% of pregnancies, the fetal risk for most medications is unknown, and the majority of medications have no data regarding safety in pregnancy.

**Objective:**

Using β-blockers as a proof-of-concept, the primary objective of this study was to assess the utility of Twitter data for a cohort study design—in particular, whether we could identify (1) Twitter users who have posted tweets reporting that they took medication during pregnancy and (2) their associated pregnancy outcomes.

**Methods:**

We searched for mentions of β-blockers in 2.75 billion tweets posted by 415,690 users who announced their pregnancy on Twitter. We manually reviewed the matching tweets to first determine if the user actually took the β-blocker mentioned in the tweet. Then, to help determine if the β-blocker was taken during pregnancy, we used the time stamp of the tweet reporting intake and drew upon an automated natural language processing (NLP) tool that estimates the date of the user’s prenatal time period. For users who posted tweets indicating that they took or may have taken the β-blocker during pregnancy, we drew upon additional NLP tools to help identify tweets that report their pregnancy outcomes. Adverse pregnancy outcomes included miscarriage, stillbirth, birth defects, preterm birth (<37 weeks gestation), low birth weight (<5 pounds and 8 ounces at delivery), and neonatal intensive care unit (NICU) admission. Normal pregnancy outcomes included gestational age ≥37 weeks and birth weight ≥5 pounds and 8 ounces.

**Results:**

We retrieved 5114 tweets, posted by 2339 users, that mention a β-blocker, and manually identified 2332 (45.6%) tweets, posted by 1195 (51.1%) of the users, that self-report taking the β-blocker. We were able to estimate the date of the prenatal time period for 356 pregnancies among 334 (27.9%) of these 1195 users. Among these 356 pregnancies, we identified 257 (72.2%) during which the β-blocker was or may have been taken. We manually verified an adverse pregnancy outcome—preterm birth, NICU admission, low birth weight, birth defects, or miscarriage—for 38 (14.8%) of these 257 pregnancies. We manually verified a gestational age ≥37 weeks for 198 (90.4%) and a birth weight ≥5 pounds and 8 ounces for 50 (22.8%) of the 219 pregnancies for which we did not identify an adverse pregnancy outcome.

**Conclusions:**

Our ability to detect pregnancy outcomes for Twitter users who posted tweets reporting that they took or may have taken a β-blocker during pregnancy suggests that Twitter can be a complementary resource for cohort studies of drug safety in pregnancy.

## Introduction

Prescription or over-the-counter medication is taken during more than 90% of pregnancies [1]. Despite the widespread use of medication during pregnancy, the fetal risk for most medications approved by the US Food and Drug Administration is unknown, and the majority of approved medications have no data regarding safety in pregnancy [2]. Given that Twitter has become a popular source of data on health conditions [3], it should be explored for evaluating drug safety in pregnancy, especially since 42% of people aged 18-29 years and 27% of people aged 30-49 years in the United States use Twitter [4]. Our prior work [5] used Twitter data in a case-control study that involved identifying users who reported a birth defect outcome (cases) [6] and users who did not (controls), and then searching their tweets for reports of medication exposure during pregnancy. Twitter data has not been assessed, however, for its utility in a cohort study design, which would involve identifying pregnancy outcomes for users who have reported taking medication during pregnancy.

Using β-blockers as a proof-of-concept, the primary objective of this study was to assess whether we could identify (1) Twitter users who have posted tweets reporting that they took medication during pregnancy and (2) their associated pregnancy outcomes, including miscarriage, stillbirth, birth defects, preterm birth (<37 weeks gestation), low birth weight (<5 pounds and 8 ounces at delivery), and neonatal intensive care unit (NICU) admission. We chose β-blockers as an example because cardiovascular disease is the leading cause of pregnancy-related deaths in the United States [7] and β-blockers are the most common type of medication for treating cardiac conditions during pregnancy [8]. Meanwhile, data on the safety of maternal β-blocker exposure are inconsistent; some studies report associations with low birth weight, preterm birth, perinatal mortality, or birth defects [9-17], while others do not [18-24].

## Methods

### Ethical Considerations

The Twitter data used in this study were collected and analyzed in accordance with the Twitter Terms of Service. The Institutional Review Board of the University of Pennsylvania reviewed this study and deemed it exempt human subjects research under 45 CFR §46.101(b)(4) for publicly available data sources (protocol# 828972). Although the tweets presented in this paper were public at the time of this study, we have slightly modified them, including removing usernames and URLs and redacting names, to help deidentify the users.

### Medication Intake

We searched for mentions of β-blockers and their lexical variants (eg, misspellings) [25] in 2.75 billion tweets posted by 415,690 users who announced their pregnancy on Twitter [26]. Table 1 provides the β-blocker keywords and their lexical variants. We used annotation guidelines [27] to manually distinguish tweets reporting that the user actually took the β-blocker. If the tweet reported intake but did not explicitly indicate that the intake occurred during pregnancy, we used the time stamp of the tweet and drew upon an automated natural language processing (NLP) tool [28] that estimates the date of the user’s prenatal time period. We also identified reports of taking a β-blocker that occurred before or after pregnancy, assuming that, if there was no evidence in the tweet that the user stopped taking it before pregnancy or started taking it after pregnancy, the user may have been taking it during pregnancy. We excluded users for whom we could not estimate the date of their prenatal time period.

**Table 1:** Keywords and their lexical variants used to search for tweets that mention β-blockers.

Keyword	Lexical variants
Acebutolol	N/A^a^
Atenolol	Atenelol, atenonol, atenanol, antenolol, atenol, atenolo, atenalol, antenenol, atentol, atenenol, attenalol, atenlol, attenolol, altenolol
Beta blocker	Beta-blocker, b blocker, b-blocker, beta blockers, beta-blockers, b blockers, b-blockers, betablocker, bblocker, betablockers, bblockers
Carvedilol	Carvidolol
Coreg	N/A
Corgard	N/A
Inderal	Inderall, inderol
Labetalol	Labetolol
Lopressor	N/A
Metoprolol	Metopolol, metropolol, metorolol, metroprolol, metaprolol, metoporol, metprolol, metotoprolol, metropolo, metroplol, meteprolol, metoprol, metroporol, metoprolo
Nadolol	Nadalol
Normodyne	N/A
Propranolol	Propananol, propanonol, proprapanol, propranonol, proranolol, propanolol, propranalol, proprananol, propanalol, propronolol
Sectral	N/A
Trandate	N/A
Tenormin	N/A
Toprol	Toprol, toprolol, topral, toperol, tropol, toporal, toporol, toporolol

^a^N/A: not applicable.

### Pregnancy Outcomes

For users who posted tweets indicating that they took or may have taken the β-blocker during pregnancy, we drew upon automated NLP tools [29,30] to help identify tweets that self-report an associated pregnancy outcome, including miscarriage, stillbirth, birth defects, preterm birth, low birth weight, and NICU admission. To assess a potential reporting bias, we drew upon an automated NLP tool [31] that detects tweets reporting a gestational age ≥37 weeks (indicates the lack of miscarriage and preterm birth) or a birth weight ≥5 pounds and 8 ounces (indicates the lack of low birth weight, miscarriage, and stillbirth). If we did not automatically detect a tweet explicitly reporting a gestational age ≥37 weeks, we manually analyzed tweets posted during this time for evidence that the user was still pregnant.

### Covariates

Two important potential confounders when evaluating drug safety in pregnancy are maternal age and indication for use. To help identify maternal age, we deployed an automated NLP tool [32] that identifies tweets self-reporting the exact age of the user at the time the tweet was posted. Then, we used the date of the user’s prenatal time period to determine the user’s age during pregnancy. To identify an indication for use, we manually reviewed the tweets reporting intake of a β-blocker posted by users who took or may have taken the β-blocker during pregnancy.

## Results

Excluding retweets, we retrieved 5114 tweets, posted by 2339 users, that mention a β-blocker, and manually identified 2332 (45.6%) tweets, posted by 1195 (51.1%) of the users, that self-report taking the β-blocker. We were able to estimate the date of the prenatal time period for 334 (27.9%) of the 1195 users. Because some users’ collection of tweets span several years and include multiple pregnancies, we identified 356 pregnancies among these 334 users. Among these 356 pregnancies, we found evidence that a β-blocker was taken during 58 (16.3%) of them and may have been taken during 199 (55.9%) of them. Table 2 presents examples of two users’ tweets. User 1 reported on January 25, 2020, that the baby’s due date was in 100 days, so our automated tool [28] estimated that pregnancy began on July 29, 2019, and would end on May 4, 2020. On April 16, 2020, User 1 explicitly reported taking Propranolol during pregnancy. User 1 reported that the baby was born premature on April 2, 2020—between 35 and 36 weeks gestation—with a low birth weight of 4 pounds and 12 ounces, and was admitted to the NICU. User 2 reported being 37 weeks pregnant on June 1, 2020, so our automated tool [28] estimated that pregnancy began on September 16, 2019, and would end on June 22, 2020. Whereas User 1 explicitly reported taking a β-blocker during pregnancy, for User 2, we used the time stamp of March 26, 2020, to infer that the intake was during pregnancy. User 2 reported on June 11, 2020, that the baby was born—between 38 and 39 weeks gestation—and weighed 7 pounds and 5 ounces at birth.

We manually verified an adverse pregnancy outcome—preterm birth, NICU admission, low birth weight, birth defects, or miscarriage—for 38 (14.8%) of the 257 pregnancies during which a β-blocker was or may have been taken. Table 3 presents the adverse pregnancy outcomes among these 257 pregnancies. We manually verified a gestational age ≥37 weeks for 198 (90.4%) and a birth weight ≥5 pounds and 8 ounces for 50 (22.8%) of the 219 pregnancies for which we did not identify an adverse pregnancy outcome. We identified maternal age for 222 (86.4%) of the 257 pregnancies during which a β-blocker was or may have been taken. Table 3 includes the mean age per adverse pregnancy outcome. We identified an indication for taking the β-blocker for 197 (76.7%) of these 257 pregnancies—for example, tachycardia, hypertension, anxiety, and migraines.

**Table 2:** Sample tweets used to determine exposure to β-blockers during pregnancy and associated pregnancy outcomes.

User and tweet		Time stamp	Pregnancy start	Pregnancy end
**1**			2019-07-29	2020-05-04
	exactly 100 days til my due date!	2020-01-25		
	I was on Propranolol during my pregnancy and I had the CRAZIEST dreams I swear	2020-04-16		
	Officially introducing [name], born April 2nd, 2020. 4lbs 12oz, 18”. She’s in NICU due to being premature, but she’s doing well!	2020-04-03		
**2**			2019-09-16	2020-06-22
	5yo called me fat after I told 2.5yo I was too large to fit between their seats because of the baby. #37weekspregnant	2020-06-01		
	I saw the MFM and cardiologist last week. It was determined my cardiomyopathy is manageable and I was put on a beta blocker	2020-03-26		
	Introducing [name] 7lbs 5oz 20” long Csection went really well. We can’t wait until the big boys get to meet him	2020-06-11		

**Table 3:** Self-reported adverse pregnancy outcomes for Twitter users who took or may have taken a β-blocker during pregnancy (N=257).

Pregnancy outcome	Number of users, (%)	Sample tweet	Mean age
Preterm birth	23 (8.9%)	[name] came at 35 weeks. My baby is small, even for a preemie.	29 (n=20)
Neonatal intensive care unit admission	12 (4.7%)	Our sweet girl has been in the NICU these past few days. She's doing better everyday and we're really hoping she gets to go home soon.	27 (n=10)
Low birth weight	9 (3.5%)	Officially introducing [name], born April 2nd, 2020 at 11:01am. 4lbs 12oz, 18 inches.	27 (n=9)
Birth defect	4 (1.6%)	My son was also born with Craniosynostosis (Sagittal). He’s now 4 and wears his ‘wiggly’ line with pride	27 (n=3)
Miscarriage	1 (0.4%)	One of the worst parts of #miscarriage is ur 1st period afterwards. It’s so definitive, so confirming that it’s over #babyloss	45 (n=1)
Stillbirth	0 (0%)	N/A^a^	N/A
Composite^b^	38 (14.8%)	N/A	28 (n=33)

^a^N/A: not applicable.

^b^Multiple adverse pregnancy outcomes were identified for some pregnancies, so the number of composite adverse pregnancy outcomes is less than the sum of the individual adverse pregnancy outcomes.

## Discussion

### Principal Findings

Our ability to detect pregnancy outcomes for Twitter users who posted tweets reporting that they took or may have taken a β-blocker during pregnancy suggests more generally that Twitter could be a complementary resource for cohort studies of drug safety in pregnancy. Additionally, our ability to identify both the maternal age and indication for taking a β-blocker for many of the users demonstrates that Twitter data would even allow such studies to account for the effect of these two important potential confounders. This study suggests that Twitter data may be particularly valuable for assessing associations with preterm birth, given both the volume of its reports on Twitter and our finding that preterm birth is largely unaffected by a potential reporting bias; that is, we detected a gestational age ≥37 weeks for 198 (90.4%) of the 219 pregnancies for which we did not identify an adverse pregnancy outcome.

### Limitations

Low birth weight may be affected by a potential reporting bias, given that we detected a birth weight ≥5 pounds and 8 ounces for only 50 (22.8%) of these 219 pregnancies. Although the rate of miscarriage in the United States is upward of more than 20% [33], our detection of miscarriage may be limited by a selection bias if users tend to announce their pregnancy on Twitter at a gestational age after which miscarriage infrequently occurs. Given our initial sample of 257 users, it is not surprising that we did not detect any reports of stillbirth, which has an incidence of <1% in the United States [34]. Nonetheless, our prior work [30] demonstrates that users do report stillbirth outcomes on Twitter, and our identification of users announcing their pregnancy on Twitter continues to grow in real time [26].

### Conclusions

Given the widespread use of medication during pregnancy and the insufficient data on fetal risks, Twitter can be a complementary resource for cohort study designs.

## Abbreviations

NICU: neonatal intensive care unit
NLP: natural language processing
